# Identification and molecular characterization of a metagenome-derived L-lysine decarboxylase gene from subtropical soil microorganisms

**DOI:** 10.1371/journal.pone.0185060

**Published:** 2017-09-20

**Authors:** Jie Deng, Hua Gao, Zhen Gao, Huaxian Zhao, Ying Yang, Qiaofen Wu, Bo Wu, Chengjian Jiang

**Affiliations:** 1 State Key Laboratory for Conservation and Utilization of Subtropical Agro-bioresources, College of Life Science and Technology, Guangxi University, Nanning, Guangxi, China; 2 College of Ocean and Biotechnology, Guangxi University for Nationalities, Nanning, Guangxi, China; Dong-A University, REPUBLIC OF KOREA

## Abstract

L-lysine decarboxylase (LDC, EC 4.1.1.18) is a key enzyme in the decarboxylation of L-lysine to 1,5-pentanediamine and efficiently contributes significance to biosynthetic capability. Metagenomic technology is a shortcut approach used to obtain new genes from uncultured microorganisms. In this study, a subtropical soil metagenomic library was constructed, and a putative LDC gene named *ldc1E* was isolated by function-based screening strategy through the indication of pH change by L-lysine decarboxylation. Amino acid sequence comparison and homology modeling indicated the close relation between Ldc1E and other putative LDCs. Multiple sequence alignment analysis revealed that Ldc1E contained a highly conserved motif Ser-X-His-Lys (Pxl), and molecular docking results showed that this motif was located in the active site and could combine with the cofactor pyridoxal 5′-phosphate. The *ldc1E* gene was subcloned into the pET-30a(+) vector and highly expressed in *Escherichia coli* BL21 (DE3) pLysS. The recombinant protein was purified to homogeneity. The maximum activity of Ldc1E occurred at pH 6.5 and 40°C using L-lysine monohydrochloride as the substrate. Recombinant Ldc1E had apparent *K*_m_, *k*_cat_, and *k*_cat_/*K*_m_ values of 1.08±0.16 mM, 5.09±0.63 s^−1^, and 4.73×10^3^ s^−1^ M^−1^, respectively. The specific activity of Ldc1E was 1.53±0.06 U mg^−1^ protein. Identifying a metagenome-derived LDC gene provided a rational reference for further gene modifications in industrial applications.

## Introduction

L-Lysine decarboxylase (LDC, EC 4.1.1.18) catalyzes the decarboxylation of L-lysine to 1,5-pentanediamine (also known as cadaverine and pentamethylene diamine) with pyridoxal 5′-phosphate (PLP) as a coenzyme [1]. This reaction is the initial and rate-limiting step in the synthesis of 1,5-pentanediamine, an important chemical precursor for industrial polymers such as polyamides and nylon [2,3]. 1,5-Pentanediamine produces nylon-5.10, which is lighter and has lower water absorption and better dimensional stability than nylon-6 [4,5]. In addition, 1,5-pentanediamine can be used to prepare desferrioxamine B for the treatment of iron overload and pathological iron deposition in humans [6]. 1,5-Pentanediamine is a cell wall component that maintains normal bacterial growth in some Gram-negative bacteria, such as *Selenomonas ruminantium* and *Anaerovibrio lipolytica* [7–9]. Therefore, 1,5-pentanediamine is important in the synthesis of high-value compounds in industrial and pharmacological settings.

The current production of 1,5-pentanediamine is mainly through traditional chemical synthesis. Although the synthetic process yields 1,5-pentanediamine at a relatively low cost, the method uses non-renewable fossil fuels and produces serious pollutants in the environment [10]. Microbial fermentation uses renewable resources but is not broadly applicable in producing 1,5-pentanediamine because of the low efficiency of LDC [11]. LDC activity sharply reduces when the pH of the medium increases from the consumption of a proton [12]. The change in pH greatly influences LDC activity. Currently, a constitutive enzyme LdcC and an acid-inducible enzyme CadA from *Escherichia coli* are commonly reported. LdcC is more active in vitro under a broad pH range than CadA, but the expression level of the latter is higher than that of the former [13]. *Corynebacterium glutamicum* and *E*. *coli* are used to construct 1,5-pentanediamine-producing strains, especially *C*. *glutamicum*, which is not synthesized LDC by itself but is widely used to produce L-lysine in industrial applications [14–18]. Overexpression of the *cadA* gene in *C*. *glutamicum* produces 1,5-pentanediamine at a concentration of 2.6 g L^−1^ [16]. The *ldcC* gene combined with the strong promoter *tuf* is overexpressed in *C*. *glutamicum*, and the yield is 300 mM M^−1^ glucose with the addition of the cofactor PLP [15]. A recent study has reported that LDC from other sources, such as *Klebsiella oxytoca* [19] and *Hafnia alvei*, can also produce 1,5-pentanediamine [20]. The *ldc* gene from *K*. *oxytoca* was expressed with the *tac* promoter in *E*. *coli*, and the conversion yield of lysine-HCl to 1,5-pentanediamine was 92% without generating side products [19]. The *ldc* gene from *H*. *alvei* was expressed in *E*. *coli* JM109 and subjected to directed evolution to obtain a mutant strain. The mutant strain produced 1,5-pentanediamine at a concentration of 63.9 g L^−1^, which was 5.6% higher than that generated by the original strain [20]. The characteristics of LDC from *K*. *pneumoniae* and *H*. *alvei* have been reported. The optimum pH of LDC from *K*. *pneumoniae* in vitro is 6.0 [21]. The LDC from *H*. *alvei* has an optimum pH of 6.5, and the specific enzyme activity is higher than that of CadA and LdcC from *E*. *coli* [20]. Hence, discovering new LDC characteristics for the construction of highly efficient engineering strains is significant.

In the microbial community, only 1% or fewer microorganisms can be cultured in a medium [22,23], while the rest are uncultivable [24]. Metagenomics has been an advanced methodology, which involves successful extraction of whole genomic DNA from the environment, construction of metagenomic libraries, and screening for novel functional genes and/or biologically active compounds [25,26]. However, few studies on the identification of novel LDC from uncultured environmental samples were reported.

In the current study, a plasmid metagenomic library was constructed from subtropical soil microorganisms. The gene encoding of a putative LDC, Ldc1E, was isolated and identified through functional and sequence-based screenings of the metagenomic DNA library. The derived amino acid sequence exhibited moderate similarity to the ornithine decarboxylase family and the aminotransferase (AAT) superfamily. This research will take an insight into the novel L-lysine decarboxylase from uncultured soil microorganisms. Moreover, gene cloning and functional identification of a metagenome-derived LDC provide novel materials for 1,5-pentanediamine biosynthesis.

## Materials and methods

### Ethics statement

The study was approved by the insititutional review board of the State Key Laboratory for Conservation and Utilization of Subtropical Agro-bioresources, College of Life Science and Technology, Guangxi University, China. Subtropical soil sample collection in this study did not require permission from relevant authorities.

### Plasmid, strains, and culture conditions

*E*. *coli* DH5α (Novagen) and pGEM-3Zf(+) (Promega) served as the host and the vector for the construction of a subtropical soil metagenomic library, respectively. Plasmid pET-30a(+) (Novagen) and bacterial strain *E*. *coli* BL21 (DE3) pLysS (Novagen) were used as the expression vector and host, respectively. The *E*. *coli* strains were grown at 37°C in Luria–Bertani (LB) medium. Restriction enzymes and ligases were purchased from ThermoFisher. *FastPfu* DNA polymerase and related reagents were purchased from TransGen Biotech, Beijing, China, and 1,5-pentanediamine, acetonitrile, acetone, and dansyl chloride were obtained from Sigma–Aldrich. A BCA Protein Assay Kit was purchased from Solarbio, China. The rest of the chemicals were analytical grade.

### Construction of a metagenomic library

Soil samples were collected from the ground surface in mountainous areas located in Guangxi, South China (N22°54′8.20″, E108°20′18.85″). The collected soil samples were maintained at 30°C and pH 6.5 and covered with rotten leaves for several years. Metagenomic DNA was extracted from subtropical soil samples following the method described by Bürgmann with modifications [27]. Purified soil DNA was partially digested with *Pst*I and *Hind*III, and the DNA fragments were examined in 0.8% agarose gel. Fractions containing 2.0–10.0 kb DNA were ligated into the pGEM-3Zf(+) vector, and the ligated products were transformed to competent *E*. *coli* DH5α. The constructed metagenomic library was collected in 96-well plates and then stored at −80°C.

### Library screening and ldc1E gene isolation

Metagenomic library clones were cultured onto screening agar plates (1% w/v tryptone, 0.5% w/v yeast extract, 1% w/v NaCl, 0.5% w/v L-lysine-HCl, 0.0016% w/v bromocresol purple, 1.5% w/v agar, pH 5.5) supplemented with 50 μg mL^−1^ ampicillin at 37°C for less than 24 h. The appearance of a purple clearance zone characterized the clones with LDC activity [28]. Positive clones with LDC activity were selected, and the plasmids were sequenced.

### DNA sequence analysis and gene structure characterization

Protein translation was performed using the translation tool from the Expasy homepage [29] (http://web.expasy.org/translate/). Sequence similarities and conserved domain search were performed in the National Center for Biotechnology Information (http://www.ncbi.nlm.nih.gov/). Multiple sequence alignment was conducted with Align X in Vector NTI (InforMax, North Bethesda, MD, USA). A phylogenetic tree was generated with Molecular Evolutionary Genetics Analysis (MEGA 6.0) using the neighbor-joining method (Tempe, AZ, USA). The secondary structure was predicted using the PSIPRED Server [30] (http://bioinf.cs.ucl.ac.uk/psipred/). Protein homology modeling structure was constructed with the GalaxyWEB server [31] (http://galaxy.seoklab.org/). The protein model quality was precisely evaluated using MOLPROBITY [32] (http://molprobity.biochem.duke.edu/). The protein model was used for docking with PLP and L-lysine in Autodock 4.2 (La Jolla, CA, USA) with default parameters. The protein structure model was displayed using PyMOL (New York, NY, USA).

### Cloning, expression, and purification of Ldc1E

The *ldc1E* nucleotide sequence was amplified from the plasmid of a positive clone. Restriction enzyme sites (underlined) for *Nco*I and *Hind*III were designed in the forward/reverse primer (5′-CTACCATGGCTAACATCATTGCCATCCTCAACC-3′/ 5′-CCCAAGCTTACGCAGCACCTTGACGGTGT-3′). Polymerase chain reaction (PCR) was performed in a 50 μL reaction consisting of 0.2 μM forward primer, 0.2 μM reverse primer, 0.2 mM dNTP, 1×*TransStart FastPfu* buffer, 2.5 U *TransStart FastPfu* DNA polymerase, and 20 ng plasmid. The PCR program was as follows: 95°C for 2 min; 30 cycles of 95°C for 20 s, 62°C for 20 s, and 72°C for 1 min; final extension step at 72°C for 5 min. The PCR product was purified and digested with *Nco*I and *Hind*III at 37°C for 2 h and then directly ligated into the *Nco*I-*Hind*III site of expression vector pET-30a(+). The confirmed recombinant plasmid pET-30a-*ldc1E* was transformed into competent *E*. *coli* BL21 (DE3) pLysS.

The transformed bacterial cell was cultivated at 37°C in LB medium with kanamycin (12.5 μg mL^−1^) and chloramphenicol (50 μg mL^−1^). When the cell density at 600 nm reached 0.6, the cells were induced with 0.8 mM isopropyl-β-D-galactopyranoside (IPTG) and further grown at 28°C for 12 h. The cells were harvested by centrifugation at 4,000 rpm for 20 min at 4°C and then washed twice with pre-cooled phosphate buffer saline (pH 7.6). The cells were lyzed by sonication, and the lysate was centrifuged at 12,000 rpm for 20 min at 4°C. Recombinant Ldc1E was purified with nickel-nitrilotriacetic acid (Ni-NTA) agarose resin and analyzed by denaturing discontinuous sodium dodecyl sulfate–polyacrylamide gel electrophoresis (SDS–PAGE). The protein concentration was determined using the BCA Protein Assay Kit.

### Product identification

The product was determined by RP-HPLC (Alliance E2695 series, Waters, USA). The HPLC system was equipped with a Waters 2475 detector and a chromatographic column (4.6 × 250 mm, 5 μm, Grace Smart RP 18). Isocratic elution with 65% acetonitrile as the mobile phase was used to separate the product, and the product was detected at 340 nm. The column temperature was maintained at 25°C. The injection volume of the sample was 10 μL, and the flow rate was 1 mL min^−1^. 1,5-Pentanediamine from the enzymatic reaction was identified through comparison with the relative retention time of the 1,5-pentanediamine standard.

The sample (0.1 mL) was mixed with 2 M sodium hydroxide (20 μL) and saturated sodium bicarbonate solution (30 μL). Dansyl chloride solution (10 mg mL^−1^, 200 μL) was added to the mixture and then incubated at 60°C for 30 min. 25% ammonium hydroxide (100 μL) was added to remove the residual dansyl chloride, and the volume of the mixture was adjusted with acetonitrile to 2 mL [33]. Finally, the mixture was filtered through 0.2 μm pore-size filters and determined by RP-HPLC.

### L-Lysine decarboxylase characterization

LDC activity was determined using 2,4,6-trinitrobenzenesulfonic acid (TNBS). This method was based on the differential solubility of lysine and 1,5-pentanediamine reaction products in toluene [34]. The reaction mixture consisted of 0.1 mM PLP, 2 mM L-lysine-HCl, 3 μg recombinant protein, and 0.2 M Na_2_HPO_4_/0.1 M citric acid (pH 6.5). The reaction mixture was incubated at 40°C for 15 min and terminated by adding 1 M K_2_CO_3_ (250 μL). The mixture was added with 9.6 mM (200 μL) TNBS and then incubated at 40°C for 5 min. Each sample was thoroughly mixed with toluene (1 mL) at top speed for 1 min. The toluene layers were separated through centrifugation at 4,000 rpm for 15 min. Heat-inactivated recombinant Ldc1E served as a negative control. The absorbance was determined at 340 nm [34]. All reactions were performed in triplicates. One unit of LDC was defined as the amount of enzyme producing 1 μmol 1,5-pentanediamine per minute under the standard assay conditions.

Temperature-dependent activity assays were performed at various temperatures from 20°C to 60°C with 5°C intervals in 0.2 M Na_2_HPO_4_/0.1 M citric acid buffer (pH 6.5). The enzyme activity obtained at the optimum temperature was used to calculate the relative percentage of activity at other temperature values. To evaluate thermostability, Ldc1E was incubated in 0.2 M Na_2_HPO_4_/0.1 M citric acid (pH 6.5) for up to 1 h at the temperature range of 20–60°C.

pH-dependent activity assays were measured in 0.2 M Na_2_HPO_4_/0.1 M citric acid pH 4.0–8.0 and 0.1 M glycine–NaOH pH 8.0–10.0 at 40°C. To estimate pH stability, Ldc1E was pre-incubated at the pH range of 4.0–9.0 at 4°C for 60 h. All reactions were performed in three independent experiments.

To investigate the effect of different PLP concentrations on Ldc1E activity, we measured the enzyme activity supplemented with 0, 0.05, 0.1, 0.5, and 1.0 mM PLP. All reactions were performed in three independent experiments.

The sensitivity of Ldc1E to metal compounds and chemical reagents was analyzed by measuring the enzyme activity supplemented with different concentrations of MgCl_2_, ZnCl_2_, CuCl_2_, CaCl_2_, CoCl_2_, BaCl_2_, SrCl_2_, CrCl_2_, MnCl_2_, NiCl_2_, FeCl_3_, AlCl_3_, ethylenediamine tetraacetic acid (EDTA), dimethyl sulfoxide (DMSO), Triton-100, Tween-20, Tween-80, or SDS. The control group did not contain a metal compound or a chemical reagent. All reactions were performed in three independent experiments.

The substrate specificity of Ldc1E was measured using the standard assay. The reaction mixture contained 2 mM L-lysine-HCl/L-arginine-HCl/L-ornithine-HCl, 0.1 mM PLP, 3 μg recombinant protein, and 0.2 M Na_2_HPO_4_/0.1 M citric acid (pH 6.5). One unit was defined as the enzyme consumption of 10 μg L-lysine-HCl/L-arginine-HCl/L-ornithine-HCl per minute under the standard assay conditions.

The kinetic parameters of purified Ldc1E were assayed by linear regression from Lineweaver–Burk plots with L-lysine HCl as the substrate at pH 6.5 and 40°C [20]. Substrate concentration ranged within 0.2–3 mM. All reactions were performed in three independent experiments.

### Nucleotide sequence accession number

The *ldc1E* nucleotide sequence was deposited in the GenBank database with the accession number KX463450.

## Results and discussion

### Construction of the metagenome library and isolation of the *ldc1E* gene

Metagenomic approaches are technically feasible for exploring novel microbial resources from various environmental samples [35]. In this study, a subtropical soil metagenome library with approximately 50,000 clones was constructed. In restriction analysis, *Pst*I and *Hind*III were used to digest randomly selected clones. DNA inserts were obtained in the range of 2.5–6.0 kb, with an average of 4.0 kb (S1 Fig). The total DNA insert of the metagenome library was estimated at more than 200.0 Mb. Functional and sequence-based screening strategies were used to screen the metagenomic library (S2 Fig). Consequently, an interesting positive clone named pGEM-520 was obtained (S3 Fig). The inserted DNA fragment of pGEM-520 contained 3,984 bp. A single open-reading frame encoding an LDC gene was obtained and named as *ldc1E*.

### Sequence analysis of Ldc1E

**Fig 1:**
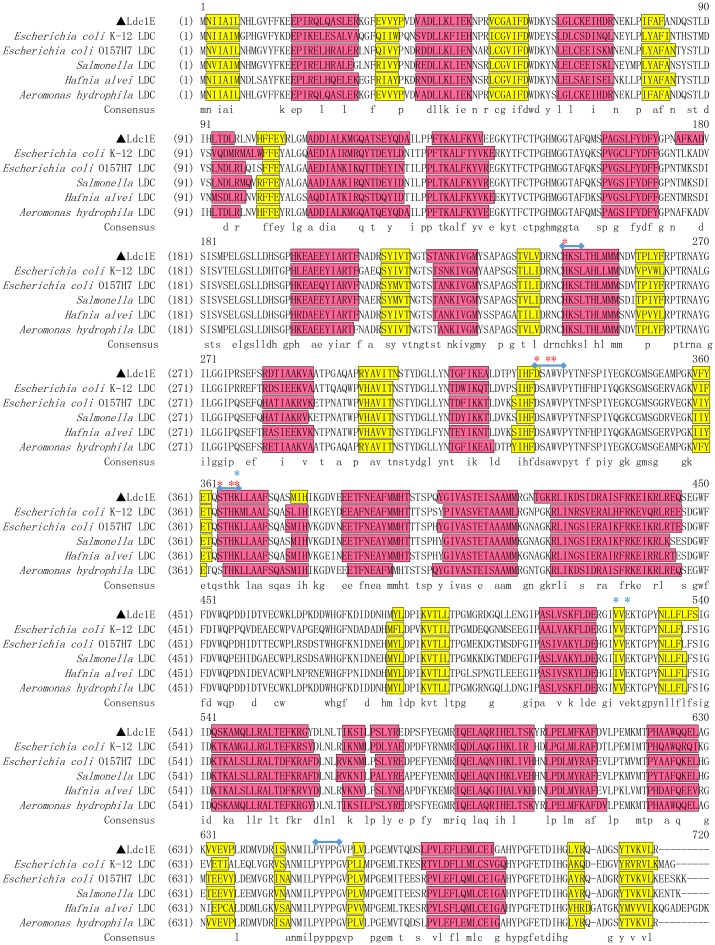
Multiple sequence alignment of the Ldc1E protein and other LDCs. Colored boxes indicate the secondary structures, which were predicted by PSIPRED Server, with helix and sheets represented by red and yellow boxes, respectively. Dashes indicate gaps. Conserved LDC decarboxylase motifs are indicated in the blue line. Red and blue asterisks show the conserved residues in PLP binding sites and the predicted residues in L-lysine binding sites, respectively. The sequences from top to bottom are *E*. *coli* K-12 LDC (P52095.2), *E*. *coli* O157:H7 LDC (P0A9H4.1), *S*. *enterica* subsp. LT2 LDC (P0A1Z0.1), *H*. *alvei* LDC (P05033.1) and *A*. *hydrophila* AL06-06 (WP_017408594.1).

Sequence analysis showed that the *ldc1E* gene consisted of 2,130 bp with an overall G+C content of 61.4% and encoded an 80.0 kDa polypeptide that has an isoelectric point of 5.43. The signal peptide was undetectable in Ldc1E. Comparison of the deduced Ldc1E sequence with the NCBI database showed that Ldc1E shared high similarities to LDC from *Aeromonas hydrophila* AL06-06 (GenBank Accession No. WP_017408594.1; 99% identity and 99% similarity) [36], which is derived from the whole genome sequencing annotation, but the function and relevant information are not verified and reported before. Ldc1E shared moderate similarities to LDC from *E*. *coli* O157:H7 (GenBank Accession No. P0A9H4.1; 76% identity and 85% similarity) [37], *Salmonella enterica* subsp. LT2 (GenBank Accession P0A1Z0.1; 74% identity and 85% similarity) [38], *H*. *alvei* (GenBank Accession No. P05033.1; 71% identity and 84% similarity) [39], and *E*. *coli* K-12 (GenBank Accession No. P52095.2; 68% identity and 82% similarity) [40]. Protein Ldc1E belonged to the AAT superfamily (fold type I) of PLP-dependent enzymes based on sequence and domain prediction. Fig 1 shows the multiple alignment of Ldc1E sequence with other LDC sequences (NCBI database). The amino acid residues related to catalytic function were highly conserved. The sequence Ser-X-His-Lys (Pxl) was highly conserved among PLP-dependent decarboxylases, and the ε-NH_3_^+^ of Lys could form a covalent bond with PLP [41]. The sequences His-Lys-Ser-Leu, Asp-Ser-Ala-Trp-Val, and Pro-Tyr-Pro-Pro-Gly were also highly conserved in LDC [42].

**Fig 2:**
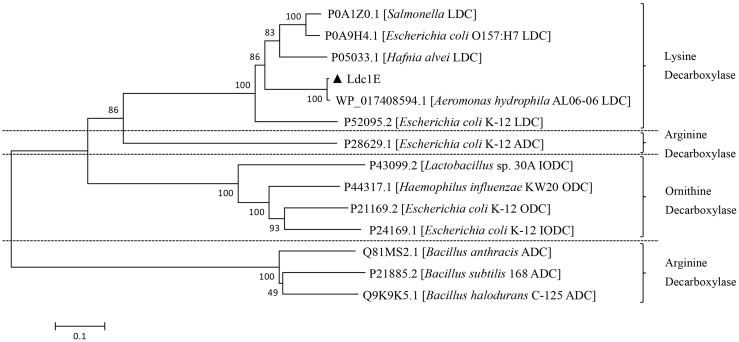
Phylogenetic tree analysis of the Ldc1E protein with other known LDCs based on the amino acid sequences. A phylogenetic tree was constructed using the neighbor-joining method with MEGA 6.0, and 1,000 bootstrap replicates were indicated at branching points. Ldc1E was shown with a solid triangle. The tree also shows the GenBank accession number and original genus of other LDCs, with the scale bar representing the number of changes per amino acid position.

A phylogenetic tree was constructed using the neighbor-joining method to show Ldc1E having a distinct clade from the LDCs of other microorganisms (Fig 2). According to the phylogenetic tree, Ldc1E and other LDCs (from *E*. *coli*, *S*. *enterica*, *H*. *alvei* and *A*. *hydrophila*) have evolved from a common ancestor. Among them, Ldc1E was closely related to *A*. *hydrophila* AL06-06 LDC. In addition, Ldc1E belonged to the same cluster as the LDC of the AAT superfamily, and other similar decarboxylases belonged to the Orn/Lys/Arg decarboxylase class-I family.

### Homology modeling and active site analysis

**Fig 3:**
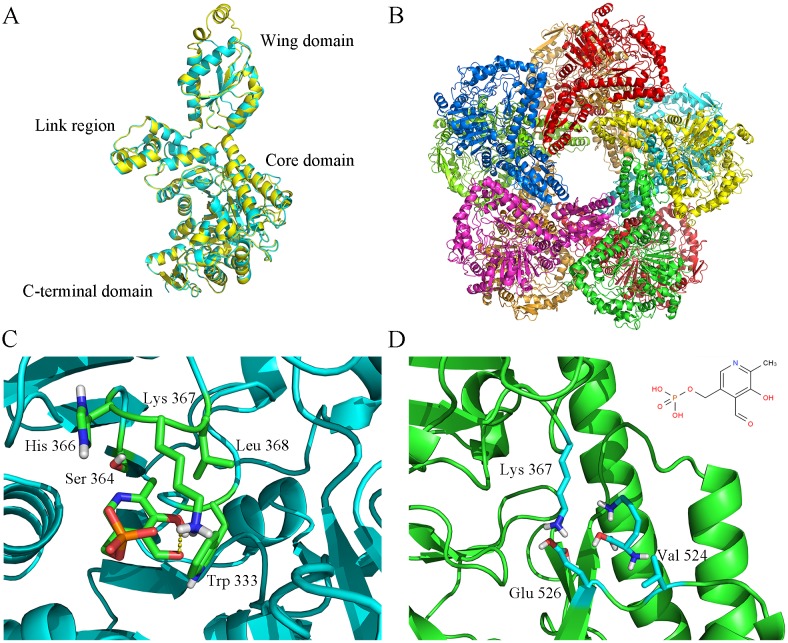
Homology modeling structure of LDC and Ldc1E. (A) Superposition of the Ldc1E monomer (yellow) on the LDC from *E*. *coli* K-12 (PDB ID: 3n75). (B) Ribbon representation of Ldc1E homo-decamer. (C) Ball-and-stick representation of the docking models of Ldc1E with PLP. Lys367 was a catalytic residue located in the active center, and the Trp333, Ser364, and His366 stabilized the PLP structure. (D) Ball-and-stick representation of the docking models of Ldc1E with L-lysine, where Lys367, Val524, and Glu526 were probably substrate binding sites.

The 3D structure model of Ldc1E was constructed by using the GalaxyWEB server, and a template (PDB: 3n75) was found by searching the amino acid sequence in Protein Data Bank [31]. 3n75 was an acid-inducible LDC from *E*. *coli* K12, and its quaternary structure was a homo-decamer [43]. The identity of Ldc1E and 3n75 was 75% with 100% coverage. Superposition of the Ldc1E monomer model onto the structure of 3n75 demonstrated their high similarities except for the wing domain (Fig 3A) [42]. The predicted quaternary structure of Ldc1E was a homo-decamer consisting of five dimers (Fig 3B). The Ldc1E monomer model had 25 α-helices and 22 β-sheets, with about 11 α-helices and 11 β-sheets in the active site. According to the SCOP structural classification of proteins, Ldc1E had a tertiary structure of α/β-fold LDC [44,45].

Docking of the Ldc1E monomer with PLP from the Autodock 4.2 software showed that the binding sites probably contained the residues Trp333, Ser364, His366, and Lys367 (Fig 3C). The Lys367 residue was highly conserved at the LDC activity site, and its ε-amino group covalently bonded to PLP as a Schiff base to form an internal aldimine [41,46]. The residues Trp333, Ser364, and His366 could also stabilize the PLP structure. Kanjee [41] reported that Ser364 and His366 residues form hydrogen bonds with the phosphate group of PLP and that the ring nitrogen NE1 of Trp333 interacts with the pyridoxal O3 oxygen atom. At present, reports on the binding sites of L-lysine substrate and LDC are scarce. The predicted L-lysine binding sites probably contained the residues Lys367, Val524, and Glu526 (Fig 3D). Gani [47] proposed that the negatively charged residue Glu526 could act as a postulated mode of substrate binding site for LDC.

### Expression and purification of the Ldc1E protein in *E*. *coli*

**Fig 4:**
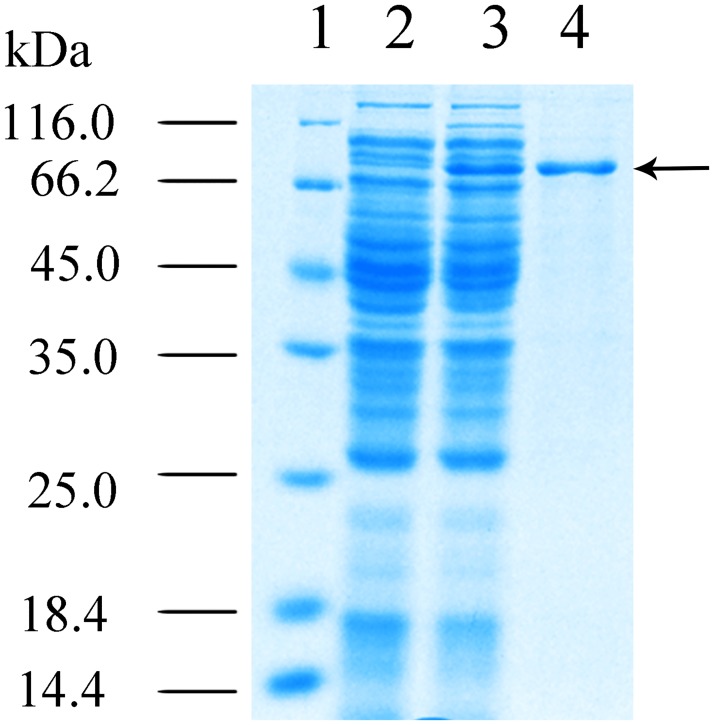
12% (w/v) SDS-PAGE analysis of recombinant Ldc1E. Lane 1, molecular mass standards. Lane 2, total protein of *E*. *coli* BL21 (DE3) pLysS harboring empty pET30a(+) (control). Lane 3, total protein of *E*. *coli* BL21 (DE3) pLysS harboring the recombinant *ldc1E* in pET30a(+). and Lane 4, protein was purified using the Ni-NTA column method. The black arrow indicates the recombinant Ldc1E.

The LDC gene *ldc1E* was subcloned into the expression vector pET30a(+) and transformed into *E*. *coli* BL21 (DE3) pLysS. Intracellular Ldc1E was expressed without any modification. The recombinant Ldc1E was expressed by induction with 0.8 mM IPTG. Crude Ldc1E was purified by Ni-NTA magnetic agarose chromatography and analyzed using SDS–PAGE. A single ~80 kDa band corresponded to the predicted size of Ldc1E (Fig 4). The molecular masses of LDCs CadA and LdcC from *E*. *coli* were 78 and 80 kDa, respectively [48], and the LDCs from *Burkholderia* sp. AIU395 and *K*. *pneumoniae* had molecular masses of 76.5 and 80 kDa, respectively [21,49]. Hence, Ldc1E had a similar molecular mass to the aforementioned LDCs.

### Functional characterization of Ldc1E

**Fig 5:**
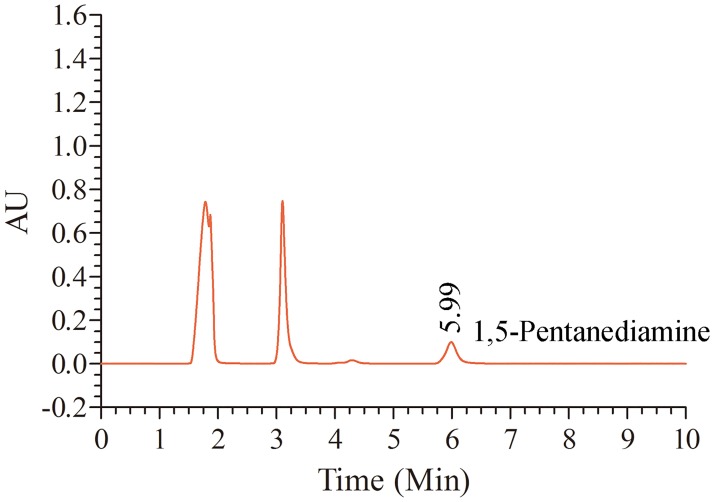
RP-HPLC profile of the reaction product from the decarboxylation of L-lysine HCl substrate under Ldc1E catalysis. The enzymatic product was derivatized with dansyl chloride.

The products of enzymatic L-lysine decarboxylation by recombinant Ldc1E were determined through RP-HPLC (Fig 5). The dansyl chloride derivative of the enzymatic product produced a peak at 5.99 min, which identically corresponded to the retention time of the 1,5-pentanediamine standard. Hence, the Ldc1E protein could catalyze the decarboxylation of L-lysine HCl to 1,5-pentanediamine.

### Effects of temperature, pH, and PLP on Ldc1E activity

**Fig 6:**
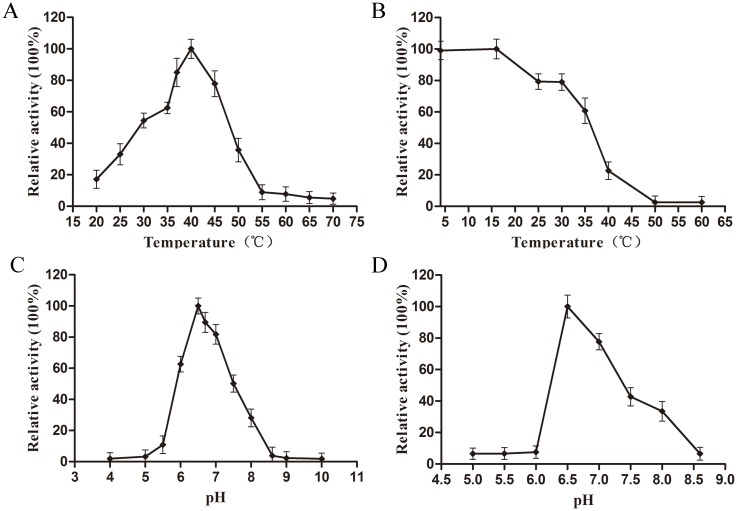
Effects of temperature and pH on the activity and stability of Ldc1E. (A) Optimum reaction temperature of the recombinant Ldc1E. The enzyme activity was measured at various temperatures from 20°C to 60°C with 5°C intervals in 0.2 M Na_2_HPO_4_/0.1 M citric acid buffer (pH 6.5). Relative activity of 100% represents the specific activity of 1.50±0.06 U mg^−1^ protein. (B) Effect of temperature on the enzymatic activity of recombinant Ldc1E. Relative activity of 100% represents the specific activity of 1.53±0.06 U mg^−1^ protein. (C) Effect of pH on the enzymatic activity of recombinant Ldc1E. The enzyme activity was measured in 0.2 M Na_2_HPO_4_/0.1 M citric acid (4.0–8.0) and 0.1 M glycine–NaOH (8.0–10.0) at 40°C. Relative activity of 100% represents the specific activity of 1.53±0.06 U mg^−1^ protein. (D) Effect of stable pH on the enzymatic activity of the recombinant Ldc1E. Relative activity of 100% represents the specific activity of 0.93±0.05 U mg^−1^ protein.

The effects of temperature and pH on the activity of recombinant Ldc1E was shown in Fig 6. The optimal temperature of Ldc1E was determined at 40°C, and the enzyme maintained 50% of its maximum activity at 30–45°C (Fig 6A). The thermostability of Ldc1E was at 4–16°C for 1 h, and the enzyme activity was 79.0% at 30°C for 1 h. Ldc1E activity rapidly decreased above 35°C, almost losing its entire activity after 1 h at 50°C (Fig 6B). Compared with other LDCs, the optimal temperature of recombinant Ldc1E was about 3°C higher than that from *K*. *pneumoniae* and *H*. *alvei* [20, 21], but about 12°C lower than that from *E*. *coli* [13]. Hence, recombinant Ldc1E was sensitive to temperature and required storage under a low temperature environment.

The optimum pH of Ldc1E was 6.5 with the enzyme displaying more than 50% of the maximum activity at the pH range of 6.0–7.5 and rapidly losing its activity outside this range (Fig 6C). The optimal pH of Ldc1E was similar to that of the LDC from *Burkholderia* sp. [49] and was between the values of CadA (pH 5.5) and LdcC (pH 7.6) in *E*. *coli* [13]. The Ldc1E was stable at pH 6.5–7.5, retaining more than 40% of the initial activity in the absence of the substrate after incubating for 60 h at 4°C (Fig 6D).

The effect of different PLP concentrations on the activity of recombinant Ldc1E was determined at pH 6.5 at 40°C (S4 Fig). The optimum supplemented PLP concentration was 0.1 mM. When the PLP concentration was 0.05 mM, Ldc1E activity was about 0.98-fold lower than that in 0.1 mM PLP. The activity of LDC from *S*. *ruminantium* with 0.05 mM PLP was about 0.66-fold lower than that of the enzyme with 0.1 mM PLP [12]. Therefore, Ldc1E probably has a higher binding affinity for PLP than LDC from *S*. *ruminantium*.

### Effects of metal ions and reagents on the activity of Ldc1E

The effects of different metal ions and chelating agents on the activity of recombinant Ldc1E were measured at pH 6.5 at 40°C (Table 1). The enzymatic reaction mixture in the absence of metal ions or chelating agents was used as a control with the enzyme activity as a baseline (100%). Among the metal ions, Mg^2+^, Cr^2+^, and Ca^2+^ had weak activating effects; Sr^2+^, Ba^2+^, Mn^2+^, and Ni^2+^ had weak inhibiting effects; and Co^2+^, Zn^2+^, Cu^2+^, Fe^3+^, and Al^3+^ had strong inhibiting effects on the enzyme activity, particularly Zn^2+^ and Cu^2+^, which have baseline values of 23.8% and 9.8%, respectively. Hence, we proposed that Ldc1E has some binding sites that combine with these metal ions to change the enzyme structure and affect the enzyme activity. EDTA and DMSO were weak activators of Ldc1E, but different concentrations of EDTA and DMSO did not significantly improve the enzyme activity. In addition, 5% TritonX-100, 5% Tween-20, 5% Tween-80, 0.5% SDS, and 1% SDS had strong inhibiting effects on the enzyme activity, with baseline values of 26.1%, 11.6%, 9.2%, 5.3%, and 5.1%, respectively.

**Table 1:** Effects of various chemicals on the recombinant Ldc1E activities.

Compound	Concentration (mM)	Relative Activity^a^ (%)
None	-	100±3.9 *
MgCl_2_	5	128.5±7.9
CrCl_2_	5	110.8±7.3
CoCl_2_	5	60.5±6.4
SrCl_2_	5	93.8±5.1
BaCl_2_	5	90.1±6.1
CaCl_2_	5	117.9±7.3
MnCl_2_	5	85.2±4.8
ZnCl_2_	5	23.8±5.1
CuCl_2_	5	9.8±4.6
NiCl_2_	5	81.2±6.6
FeCl_3_	5	61.0±5.0
AlCl_3_	5	67.9±7.1
EDTA	1	134.4±6.3
EDTA	5	120.7±7.6
EDTA	10	132.9±5.1
DMSO	5	115.9±5.5
DMSO	10	104.3±5.6
DMSO	50	125.7±10.0
TritonX-100	5%	26.1±4.9
Tween-20	5%	11.6±4.1
Tween-80	5%	9.2±3.8
SDS	0.5%	5.3±3.7
SDS	1%	5.1±3.6

^a^ Reactions were carried out at 40°C, pH 6.5, in the presence or absence (control) of the compounds listed. Activity of the control is 100%.

* The specific enzyme activity of Ldc1E without metal ion and chelating agent was 1.47±0.04 U mg^−1^ protein.

### Substrate specificity analysis

**Fig 7:**
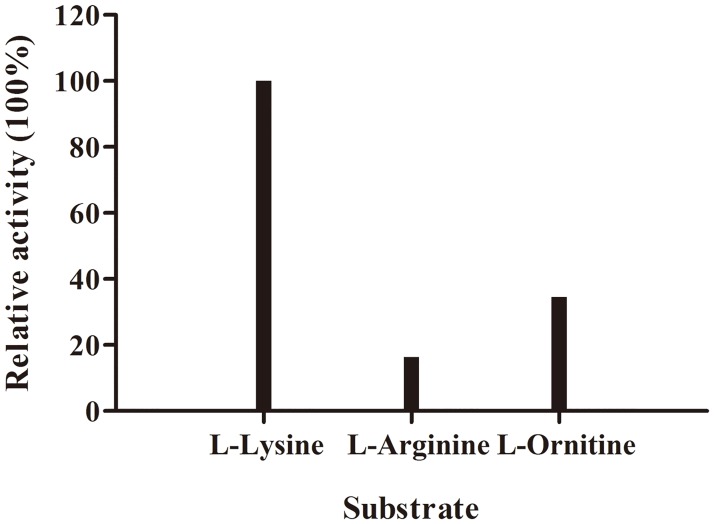
Substrate specificity test of L-lysine-HCl, L-arginine-HCl, and L-ornithine-HCl. One unit was defined as the enzyme consumption of 10 μg L-lysine-HCl/L-arginine-HCl/L-ornithine-HCl per minute, respectively.

Through substrate profile analysis, Ldc1E showed activity toward L-arginine-HCl and L-ornithine-HCl (Fig 7), but its activity was stronger toward the latter than the former. LDCs from other microorganisms had different activities toward ornithine and arginine. For instance, LDC from *K*. *pneumoniae* and *S*. *ruminantium* exhibited activity toward ornithine, whereas CadA from *E*. *coli* showed activity toward arginine [21,50].

### Enzyme kinetic analysis

Kinetic parameters of recombinant Ldc1E were analyzed by measuring the initial velocities over a range of substrate concentrations (0.2–3 mM) at pH 6.5 and 40°C. Lineweaver–Burk plots of the initial velocities determined *K*_m_ and *V*_max_ values (S5 Fig). Ldc1E had *K*_m_, *k*_cat_, and *k*_cat_/*K*_m_ values of 1.08±0.16 mM, 5.09±0.63 s^−1^, and 4.73×10^3^ s^−1^ M^−1^, respectively. These findings were comparable with the properties of various LDC from different sources (Table 2). The *K*_m_ value of LDC from *Burkholderia* was 0.84 mM, which had the best ability to combine with the L-lysine at present [49]. The *K*_m_ of Ldc1E was 4.5-fold lower than that of LDC from *H*. *alvei* and 7.1-fold lower than that of LDC from *K*. *pneumoniae* [20,21]. Thus, we considered that Ldc1E and L-lysine-HCl had a stronger binding than LDC from *H*. *alvei* and *K*. *pneumoniae*. We also demonstrated that Ldc1E had a higher catalytic activity than LDC from *H*. *alvei* and *K*. *pneumonia*, because the *k*_cat_*/K*_m_ of Ldc1E was 5.6-fold and 39-fold higher than those of LDCs from *H*. *alvei* and *K*. *pneumoniae*, respectively [20,21]. The specific enzyme activity of Ldc1E was 1.53±0.06 U mg^−1^ protein, which was higher than that from *Burkholderia* sp. AIU 395. And the specific activities of LDCs from *K*. *pneumoniae* were unreported. Furthermore, the activity of Ldc1E could undergo improvements by using rational and irrational transformation technologies to accomplish the molecular modification of the enzyme.

**Table 2:** Properties of L-lysine decarboxylase from various sources.

Sources	*K*_m_ (mM)	pH	Temperature (°C)	*k*_cat_ (s^−1^)	*k*_cat_/*K*_m_ (s^−1^ M^−1^)	References
*E*. *coli* CadA	1.5	5.5	52	None	None	Lemonnier et al. 1998 [13]
*H*. *alvei*	4.93	6.5	37	4.12	0.84×10^3^	Wang et al. 2015 [20]
*H*. *alvei* mutants	3.23	6.5	37	5.43	1.68×10^3^	
*K*. *pneumoniae*	7.7	6.0	37	0.98	0.12×10^3^	Kim et al. 2016 [21]
*Burkholderia* sp.	0.84	6.0	50	None	None	Sugawara et al. 2014 [49]
*S*. *ruminantium*	0.63	6.0	45	10.6	17×10^3^	Takatsuka et al. 1999 [50]
Subtropical soil metagenome	1.08±0.16	6.5	40	5.09±0.63	4.73×10^3^	This work

## Conclusions

A metagenome-derived LDC gene was cloned and characterized from subtropical soil microorganisms. Sequence analysis showed that the identified gene product had moderate similarities to known LDCs. The detailed biochemical characterization of the Ldc1E protein was performed, including the temperature–activity profile, pH–activity profile, metal ion–activity profile, chemical cheating activity profile, and enzyme kinetics analysis. These results indicated that Ldc1E could be a good candidate for industrial and chemical applications. Furthermore, this research also revealed the potential of using metagenomics to discover novel LDCs for converting L-lysine into 1,5-pentanediamine.

## Supporting information

S1 Fig Agarose gel electrophoresis profiles of DNA fragments. (A) Agarose gel electrophoresis profile of the metagenomic DNA. Lane 1: λ DNA/*Hind* III Marker; Lane 2: metagenomic DNA from subtropical soil microorganisms; (B) Agarose gel electrophoresis profile of the metagenomic DNA double digested with *Pst*I and *Hind*III. Lane 1: λ DNA/*Hind*III Marker; Lane 2: metagenomic DNA digested with *Pst*I and *Hind*III; (C) Agarose gel electrophoresis profile of random positive plasmid DNA from the metagenomic library. Lane 1: 1 kb DNA Marker; Lanes 2–9: recombinant plasmid digested with *Pst*I and *Hind*III; Lane 10: plasmid pGEM-3Zf(+) digested with *Pst*I and *Hind*III.
(TIF)

S2 Fig Functional screening of a metagenomic library for LDC activity. (TIF)

S3 Fig Screening of a novel LDC from the metagenomic library. Isolation of potential positive clones with functional screening strategy of LDC from the metagenomic library of subtropical soil microorganisms.
(TIF)

S4 Fig Effect of PLP concentration on Ldc1E activity. The concentrations of PLP varied at 0, 0.05, 0.1, 0.5, and 1.0 mM. LDC activity assay was performed as described in materials and methods. The specific activity of Ldc1E at 0.1 mM PLP was 1.53±0.05 U mg^−1^.
(TIF)

S5 Fig Determination of steady-state kinetics parameters, *K*_m_ and *V*_max_, for recombinant Ldc1E. The kinetic parameters of the purified enzyme Ldc1E were assayed by linear regression from Lineweaver–Burk plots with L-lysine-HCl substrate at pH 6.5 and 40°C.
(TIF)
