# Supplementary figures and images for: Identification and molecular characterization of a metagenome-derived L-lysine decarboxylase gene from subtropical soil microorganisms
Source: PLoS One. 2017 Sep 20;12(9):e0185060. doi: 10.1371/journal.pone.0185060 (PMC5607190; doi:10.1371/journal.pone.0185060)

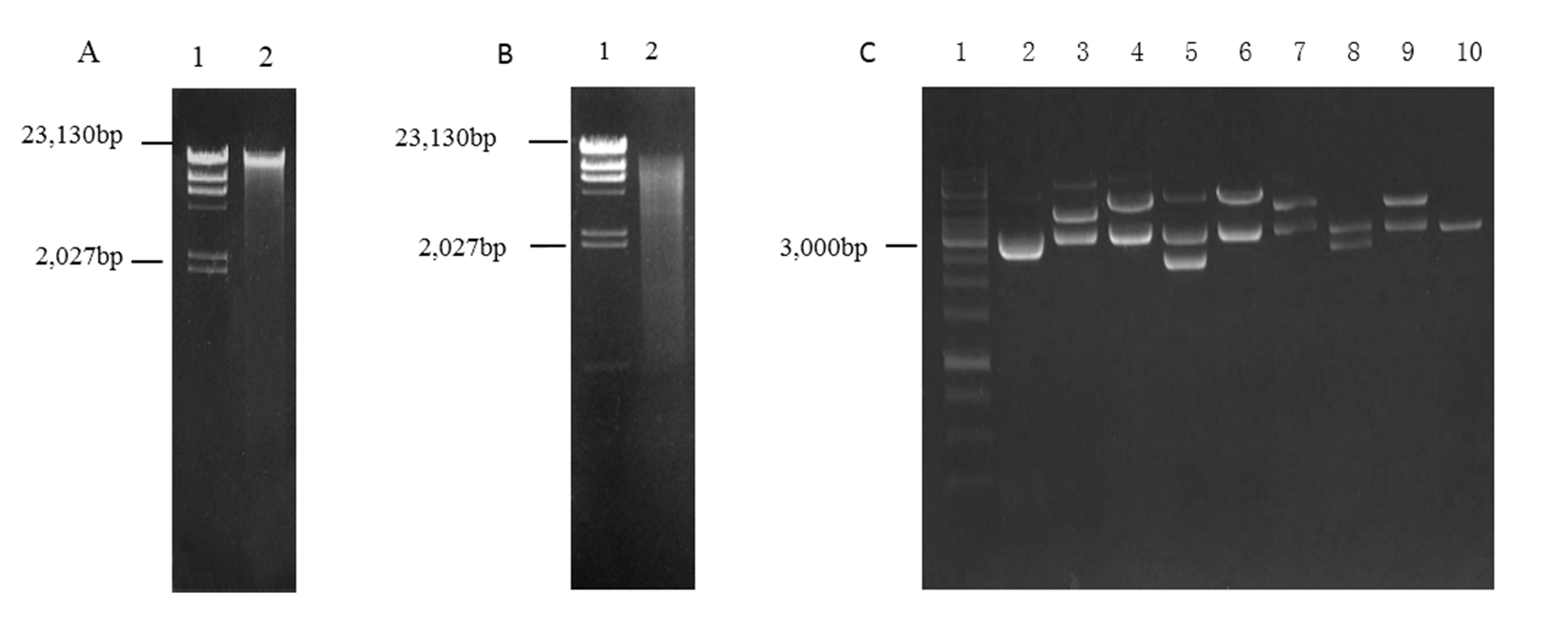

Supplement: S1 Fig — (A) Agarose gel electrophoresis profile of the metagenomic DNA. Lane 1: λ DNA/Hind III Marker; Lane 2: metagenomic DNA from subtropical soil microorganisms; (B) Agarose gel electrophoresis profile of the metagenomic DNA double digested with PstI and HindIII. Lane 1: λ DNA/HindIII Marker; Lane 2: metagenomic DNA digested with PstI and HindIII; (C) Agarose gel electrophoresis profile of random positive plasmid DNA from the metagenomic library. Lane 1: 1 kb DNA Marker; Lanes 2–9: recombinant plasmid digested with PstI and HindIII; Lane 10: plasmid pGEM-3Zf(+) digested with PstI and HindIII. (TIF) [file pone.0185060.s001.tif]

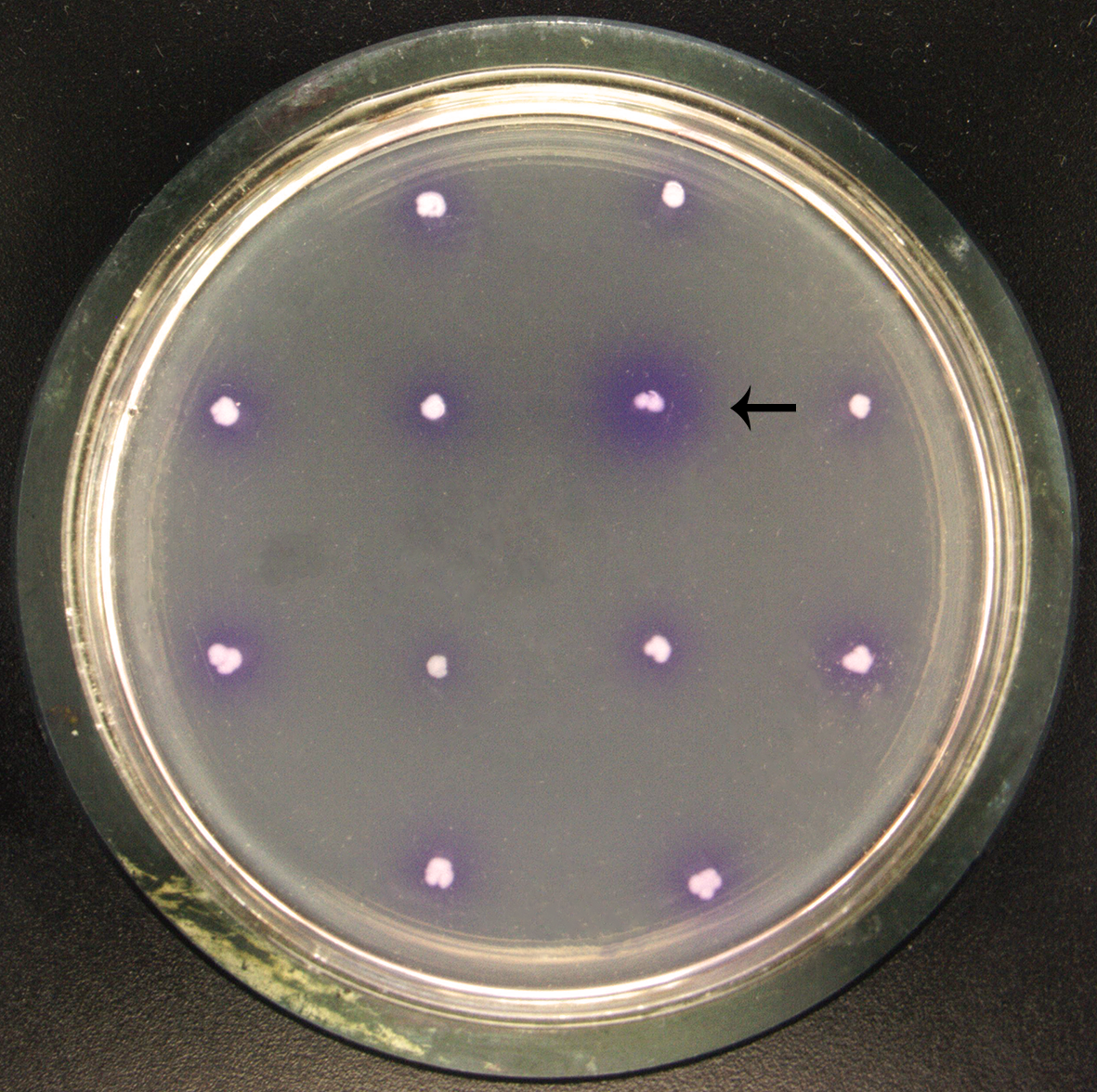

Supplement: S2 Fig — (TIF) [file pone.0185060.s002.tif]

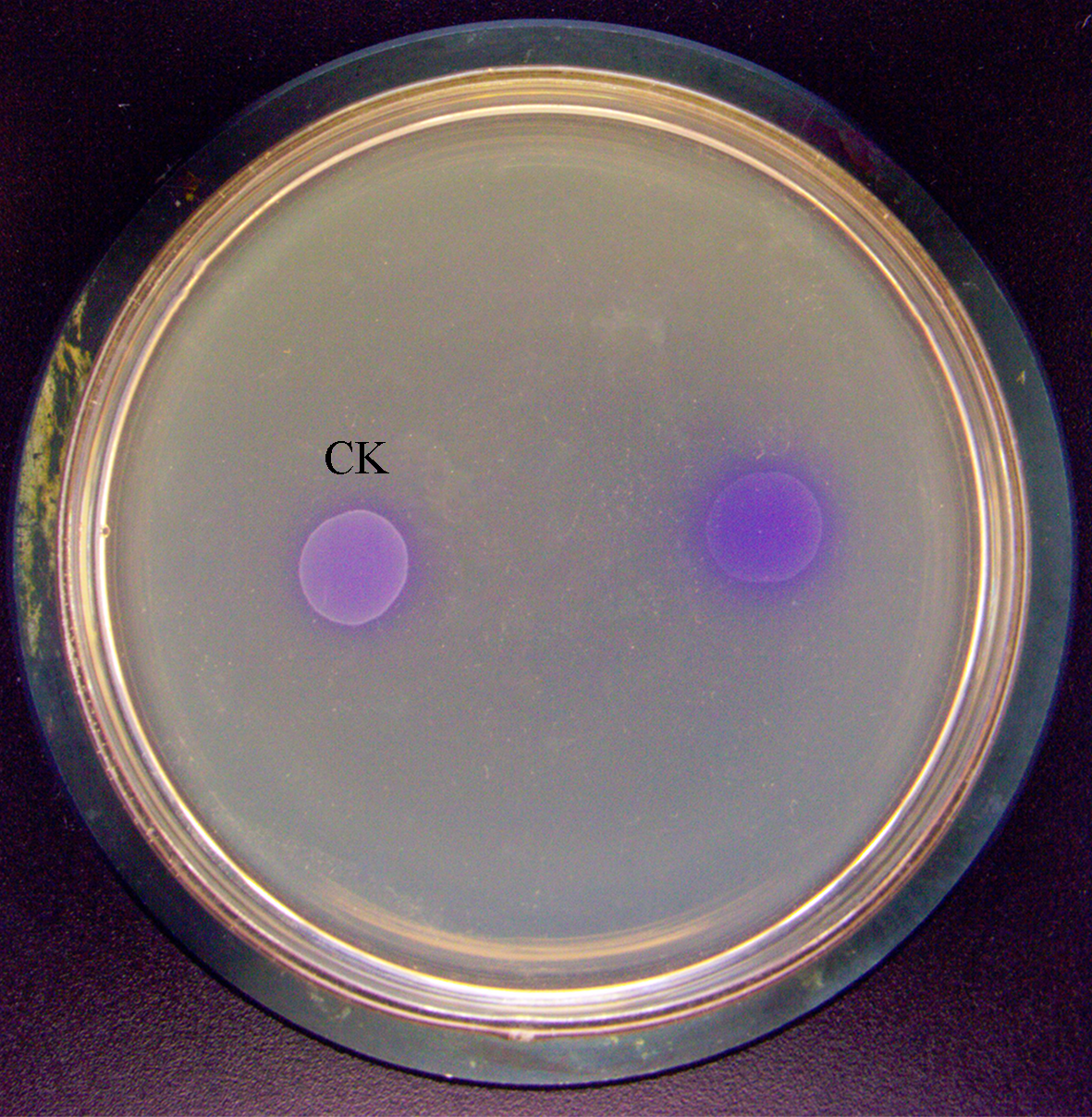

Supplement: S3 Fig — Isolation of potential positive clones with functional screening strategy of LDC from the metagenomic library of subtropical soil microorganisms. (TIF) [file pone.0185060.s003.tif]

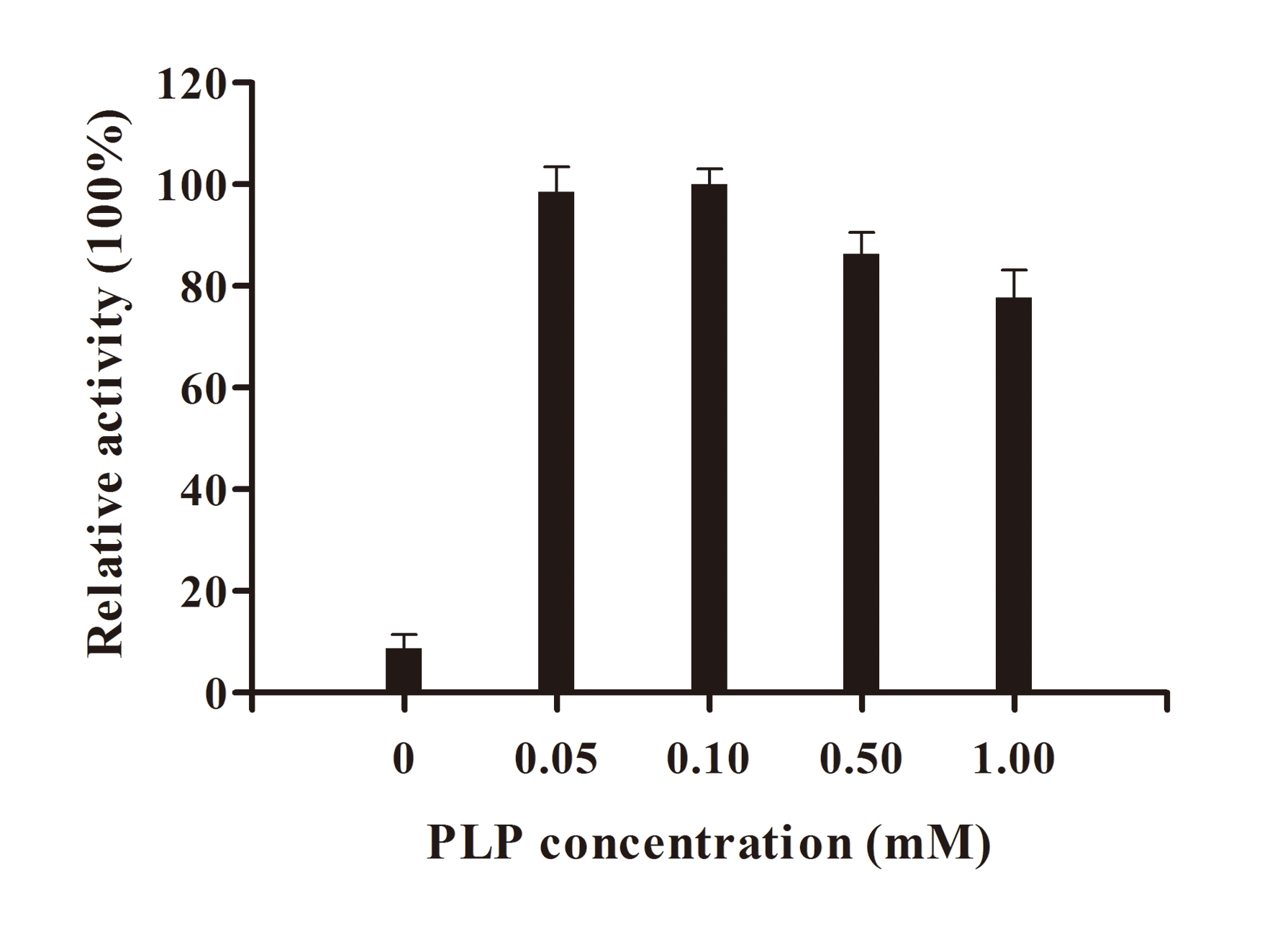

Supplement: S4 Fig — The concentrations of PLP varied at 0, 0.05, 0.1, 0.5, and 1.0 mM. LDC activity assay was performed as described in materials and methods. The specific activity of Ldc1E at 0.1 mM PLP was 1.53±0.05 U mg−1. (TIF) [file pone.0185060.s004.tif]

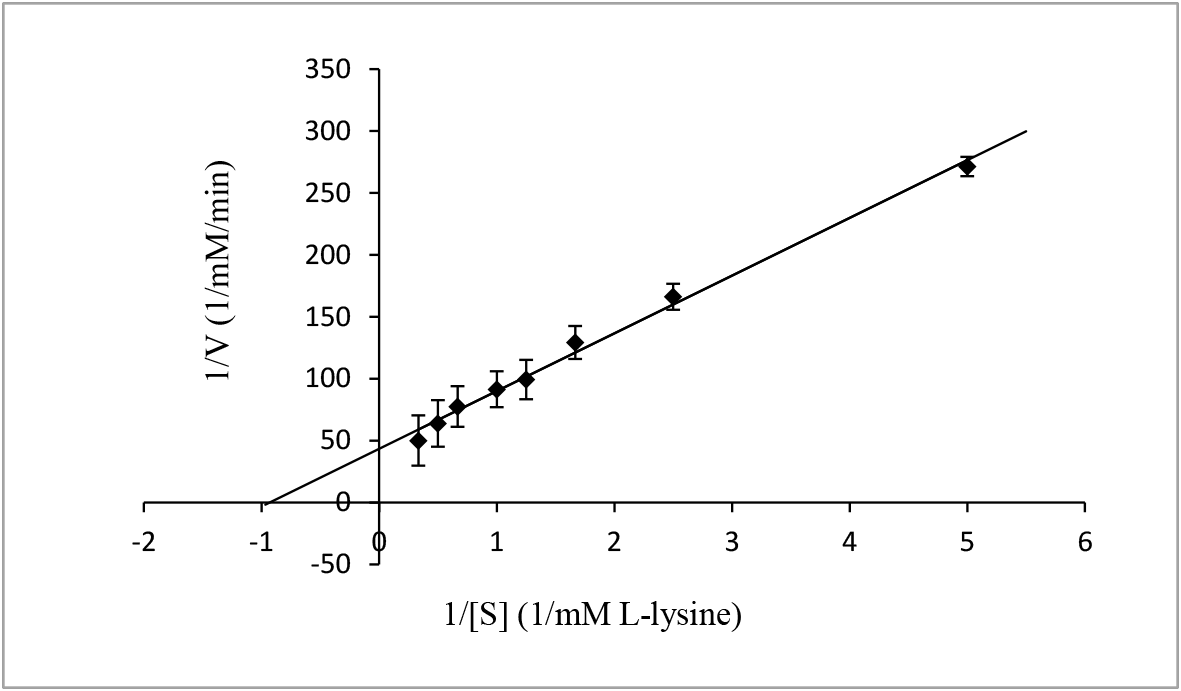

Supplement: S5 Fig — The kinetic parameters of the purified enzyme Ldc1E were assayed by linear regression from Lineweaver–Burk plots with L-lysine-HCl substrate at pH 6.5 and 40°C. (TIF) [file pone.0185060.s005.tif]
